# Weakening self-control biases the emotional evaluation of appetitive cues

**DOI:** 10.1371/journal.pone.0170245

**Published:** 2017-01-31

**Authors:** Christian Dirk Wiesner, Christoph Lindner

**Affiliations:** 1 Department of Child and Adolescent Psychiatry and Psychotherapy, Center for Integrative Psychiatry, School of Medicine, Christian-Albrechts University, Kiel, Germany; 2 Leibniz-Institute for Science and Mathematics Education (IPN) and Christian-Albrechts University, Kiel, Germany; Radboud Universiteit, NETHERLANDS

## Abstract

Exerting self-control in a first task weakens self-control in a second completely unrelated task (ego-depletion). It has been proposed that ego-depletion increases approach motivation which would amplify positive emotions to appetitive cues. Here we investigated the effect of the depletion of cognitive self-control on the subsequent emotional evaluation of appetitive cues. Participants of the depletion group copied a text omitting frequent letters and thereby exerting self-control to inhibit automated writing habits. Participants of the control group just copied the text. In a subsequent task participants had to rate valence and arousal of their responses to neutral vs. positive pictures of humans, animals, food, or sceneries. Ego-depletion caused more positive valence ratings of neutral pictures and lower arousal ratings of positive pictures. The findings do not support the notion that ego-depletion increases approach motivation in general. Rather they suggest that—without a specific motivational context—depletion of cognitive self-control differentially alters the immediate emotional evaluation of appetitive cues.

## Introduction

Self-control is the ability to override thoughts, emotions, or impulses, thus enabling humans to adapt their behavior to external demands like social norms or long-term goals [1]. Baumeister’s popular strength-model captures two striking features of self-control: Self-control seems to be domain general and to rely on a limited resource [1]. These features would explain the phenomenon of so called “ego-depletion”: Exerting self-control in a first task weakens self-control in a second completely unrelated task [2]. For example suppressing emotional reactions while watching an emotional video can weaken the ability to persist in solving anagrams [3]. Likewise, suppressing specific thoughts during writing can weaken the ability to suppress emotional reactions while watching a movie [4]. Moreover, in most studies the negative effects of ego-depletion on performance in subsequent self-control demanding tasks is not mediated by changes in mood [2–5]. In connection to recent discussions that the development of ego-depletion effects might be more complex and fragile than expected [6], several authors (e.g. [6, 7]) have suggested that the ego-depletion phenomenon needs to be explored in greater detail.

In specific, it has been criticized that the strength-model does not specify the underlying processes [7]. Accordingly, in their process-model Inzlicht et al. [7] propose that apparent failures of self-control after ego-depletion reflect a motivated shift of task priorities. This shift would enable people to balance cognitive labor to pursue ‘have-to’ goals versus cognitive leisure to pursue ‘want to’ goals [7]. One specific hypothesis about an underlying process of ego-depletion is that exerting self-control increases approach motivation [8]. This in turn should increase emotional reactions to appetitive cues like positive pictures [9]. Indeed, Wagner and colleagues report that ego-depletion increases the emotional reactivity of the amygdala to pictures [10] and the neural response to reward cues in the orbitofrontal cortex [11]. However, they do not report behavioral data of the emotional responses and the results of the first study were restricted to negative pictures. Furthermore, most behavioral studies have investigated the effect of ego-depletion on subsequent intentional suppression of emotional reactions but not on the incidental emotional reaction to stimuli (e.g. [4, 12]).

In our study we test whether the depletion of cognitive self-control increases the emotional reaction to appetitive cues. The between-subject factor (depletion vs. control) was manipulated using a text copying task. Since weak ego-depletion effects on performance might be due to unfulfilled requirements for overriding habits during the ego-depletion manipulation task [6, 13], we applied a text copying task as a well-established and valid ego-depletion manipulation [14, 15]. The emotional evaluation of appetitive cues was assessed using a picture evaluation task. We presented positive pictures of delicious food, social interactions, young animals, or beautiful sceneries as well as neutral control pictures matched for content category. We hypothesize that depletion would increase valence and arousal ratings especially of the positive pictures.

## Materials and methods

### Ethics statement

All participants gave written informed consent before the beginning of the experiment. The study protocol was discussed and approved by the research colloquium of the Leibniz-Institute for Science and Mathematics Education and by the research colloquium of the department of psychology of the University of Kiel.

### Participants

The required sample size was calculated using g*power (version 3.1.3 [16]). The expected effect size was derived from the meta-analysis by Hagger et al. [2] who reported a mean effect size of the depletion effect of Cohen’s *d* = 0.62. Muraven et al. [4] reported a study with 49 participants who were randomized to a depletion and a control condition. The dependent measures were emotional reactions during a humorous video watched after the manipulation. From the reported statistics we calculated the effect size *d*. The effect on the dependent measure “smiling” was *d* = 0.593 and the effect on “overall amusement” *d* = 0.558. In the light of recent critical papers [6, 17], we figured it would be fair to assume an effect size of at least d = 0.4 in our study. We calculated the required total sample size to detect an interaction effect in an ANOVA with a between-subject factor (*Group*: control vs. depleted) and a within-subject factor (*Target Valence*: neutral vs. positive). We assumed an alpha of .05, a minimal power of .95, and an effect size of *f* = 0.2 (*d* = 0.4). Furthermore we used a correlation coefficient of the repeated measures of *r* = .7 which we derived from other studies of our work group [18]. In fact the observed correlations were *r* = .635 for valence and *r* = .796 for arousal. According to g*power, the required total sample size is *N* = 52 to obtain a power greater than .95. Therefore our sample size of 69 should be on the save side.

Sixty-nine students (55% female) aged 24.4 ± 5.2 (mean ± SD) were recruited at a university cafeteria and were paid 5 EUR for their participation. The participants were randomly divided into a control (*n* = 34, 53.0% female) and a depletion group (*n* = 35, 57.1% female). The groups did not differ regarding gender (χ^2^ = 0.05, p = .829), handedness (χ^2^ = 2.21, p = .332), age, secondary-school grade point average (GPA), or use of recreational drugs. Further details are reported in the results section.

### Material

The self-control strength was manipulated using an ego-depletion task devised by Bertrams (e.g. [15]). Participants were asked to copy a short text describing the history of the city Mannheim. No further instructions were given to the control group. However, the depletion group was instructed to skip all instances of the letters e and n, which are the most frequent letters in German. To comply with these restrictions participants had to inhibit their well-learned, automated writing habits and write in a controlled manner. The effectiveness of the task of depleting self-control strength has been shown in several experiments (e.g. [14, 15]).

As a manipulation check we used a three-item effort scale (Cronbach’s α = .801) measuring the self-control exerted during the text copying task [14, 15]. Participants rated how strenuous and how difficult the task was and how much effort they had to put into it on three 7-point Likert scales (1 „not at all”to 7 „very much so“). To control for mood we used the Positive and Negative Affect Schedule (PANAS; German version by [19]) encompassing ten items for positive affect (e.g. „enthusiastic“) and ten items for negative affect (e.g. „nervous“) which were rated on 5-point Likert scales (1 „not at all”to 5 „extremely“). Descriptive statistics of the manipulation check are reported in Table 1.

**Table 1:** Valence and arousal ratings of the content categories.

	Neutral Mean (SEM)	Positive Mean (SEM)	*t*	*df*	*p*
*Valence*					
Humans	4.6 (0.1)	8.0 (0.2)	-19.51	68	< .001
Animals	5.8 (0.1)	7.8 (0.1)	-18.00	68	< .001
Food	5.8 (0.1)	7.2 (0.1)	-9.25	67	< .001
Sceneries	5.7 (0.1)	8.0 (0.1)	-19.86	68	< .001
*Arousal*					
Humans	2.7 (0.2)	5.1 (0.3)	-9.73	68	< .001
Animals	3.1 (0.2)	4.3 (0.2)	-6.91	68	< .001
Food	2.3 (0.2)	3.7 (0.2)	-8.89	67	< .001
Sceneries	3.0 (0.2)	5.1 (0.3)	-11.35	68	< .001

To measure the emotional evaluation of appetitive pictures we created two sets of 25 positive versus 25 neutral pictures parallelized for content categories but differing in the target valence. We selected high quality pictures from the internet that allude to certain motivational systems and are supposed to act as appetitive cues. In specific, positive pictures were chosen that allude to the affiliation motive (humans, e.g. „kissing couple“), to the motivation to care (animals, Kindchenschema, e.g. „cute duckling“), to hunger and appetite (food, e.g. „delicious cake“), or to the appreciation of beauty (sceneries, e.g. „tree by a beautiful lake“). Furthermore, for each positive picture we searched a neutral picture with similar content (e.g. humans: “couple in the office”, animals: “adult sparrow”, food: “grey-bread”, scenery: “tree by a parking lot”). The high resolution pictures were reduced to 800*600 pixels resolution, and standardized regarding luminance. Using an LCD projector, the pictures were individually presented for 2 seconds on a 3 by 2 m silver screen. After each picture participants rated their affect on the valence and arousal scales of the self-assessment manikin (SAM [20]).

As a manipulation check we compared the valence and arousal ratings of neutral and positive pictures of all four content categories (humans, animals, food, sceneries). Descriptive data and t-tests are reported in Table 1. The neutral midpoint of the valence scale was 5. The mean valence ratings of the neutral picture categories ranged from 4.6 to 5.8 (neutral). The mean valence ratings of the positive picture categories ranged from 7.2 to 8.0 (positive). Furthermore, all t-tests comparing neutral and positive pictures were highly significant (all p< .001). The arousal scale ranged from 1 (no arousal) to 9 (very high arousal). The mean arousal ratings of the neutral picture categories ranged from 2.3 to 3.1 (low arousal). The mean arousal ratings of the positive picture categories ranged from 3.7 to 5.1 (low to medium arousal). However, all t-tests comparing neutral and positive pictures were highly significant (all p< .001). Therefore, we are confident that the neutral pictures were actually neutral and the positive pictures were actually positive.

### Procedure

Participants were randomly divided in ten small groups (5 control, 5 depletion) and seated at least two seats apart in a lecture room in front of the silver screen. After answering demographic questions, the manipulation and manipulation check were administered. Finally the pictures were presented in a pseudorandom order with target valence and content categories evenly distributed over the whole set and valence and arousal were rated.

### Data analysis

Mixed design ANOVAs with *Target-Valence* (neutral vs. positive) as within-factor and *Group* (control vs. depletion) as between factor with subsequent *t*-tests were used to analyze the valence and arousal ratings of the pictures. To rule out that the results have been influenced by mood, we computed additional ANCOVAs using the positive and negative affect scales as covariates. To check whether the effect of ego-depletion was restricted to a single content category (e.g. food stimuli) we computed additional ANOVAS using *Content* category as another within-subject factor. In specific, mixed design ANOVAs of the valence and arousal ratings with the within-subject factors *Target-Valence* (neutral vs. positive) and *Content* (humans vs. animals vs. food vs. sceneries) and the between-subject factor *Group* (control vs. depletion) were computed.

## Results

The manipulation check confirmed that the depletion group experienced the text copying task as a more strenuous, difficult, and demanding effort than the control group (*t*_67_ = -5.90, *p*< .001). Still, the groups did not differ regarding positive or negative mood after the manipulation (positive affect scale: *t*_67_ = -1.05, *p* = .298; negative affect scale: *t*_67_ = -0.85, *p* = .400).

**Fig 1:**
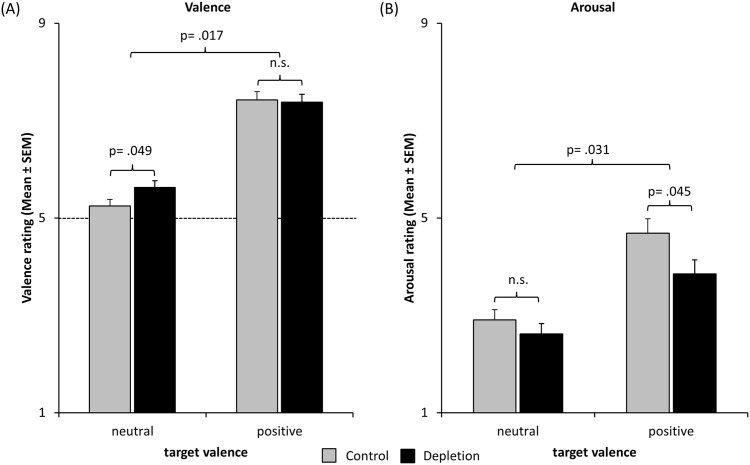
Influence of self-control depletion on the emotional evaluation of pictures. (A) The valence scale ranged from 1 „negative”thru 5 „Neutral”to 9 „positive“. (B) The arousal scale ranged from 1 „very low arousal”to 9 „very high arousal“. Valence and arousal were rated using the self-assessment manikin. The *p*-values of the lower brackets correspond to *t*-tests. The *p*-values of the higher brackets correspond to the interaction of *Group* and *Target-Valence* in an ANOVA.

Does ego-depletion bias the emotional evaluation of pictures? Indeed the valence and arousal ratings were differentially biased by the depletion manipulation (see Fig 1 and Table 2). An ANOVA of the valence ratings revealed an interaction of *Target-Valence* and *Group* (*F*_1;67_ = 5.96, *p* = .017). Subsequent *t*-tests showed that ego-depletion elicited higher valence ratings of neutral pictures (*t*_67_ = -2.00, *p* = .049) but not of positive pictures (*t*_67_ = 0.22, *p* = .825; see Fig 1A). Furthermore, a highly significant main effect of *Target-Valence* confirmed that the positive pictures were perceived as more positive than the neutral ones (*F*_1;67_ = 484.20, *p*< .001). This was true in the control group (*t*_33_ = -17.25, *p*< .001) as well as in the depletion group (*t*_34_ = -13.87, *p*< .001). There was no main effect of *Group* (*F*_1;67_ = 0.74, *p* = .393).

**Table 2:** Comparison of groups.

	ControlMean (SEM)	DepletionMean (SEM)	*t*	*df*	*p*
*Participant characteristics*					
Age	23.6 (0.6)	25.2 (1.1)	-1.31	67	.193
Secondary-school GPA	2.2 (0.1)	2.5 (0.1)	-1.36	52*	.181
Recreational drugs	0.65 (0.13)	0.63 (0.16)	0.09	67	.928
*Manipulation check*					
Effort scale	3.0 (0.2)	4.8 (0.2)	-5.90	67	**< .001**
Positive affect scale	26.4 (1.2)	28.1 (1.0)	-1.05	67	.298
Negative affect scale	12.9 (0.5)	13.7 (0.8)	-0.85	67	.400
*Dependent measures*					
Valence of neutral pictures	5.2 (0.1)	5.6 (0.1)	-2.00	67	**.049**
Valence of positive pictures	7.4 (0.2)	7.4 (0.1)	0.22	67	.825
Arousal of neutral pictures	2.9 (0.2)	2.6 (0.2)	0.96	67	.342
Arousal of positive pictures	4.7 (0.3)	3.9 (0.3)	2.04	67	**.045**

* only 54 participants entered university with the German secondary-school diploma (Abitur)

An ANOVA of the arousal ratings revealed an interaction of *Target-Valence* and *Group* as well (*F*_1;67_ = 4.89, *p* = .031). Ego-depletion lowered the arousal ratings of positive pictures (*t*_67_ = 2.04, *p* = .045) but not of neutral pictures (*t*_67_ = 0.96, *p* = .342; see Fig 1B). Again a highly significant main effect of *Target-Valence* confirmed that positive pictures were rated as more arousing than neutral ones (*F*_1;67_ = 145.78, *p*< .001; control group: *t*_33_ = -9.72, *p*< .001; depletion group: *t*_34_ = -7.26, *p*< .001). Also there was no significant main effect of *Group* (*F*_1;67_ = 2.79, *p* = .100).

Note that the differential effects of *Target-Valence* and *Group* on valence (*F*_1;65_ = 6.08, *p* = .016) and arousal (*F*_1;65_ = 4.34, *p* = .041) remain the same when we use the positive and negative affect scales as covariates.

Moreover, in additional analyses using *Content* categories as a within-factor we did not find any interactions of *Content* categories with *Group*: An ANOVA of the valence ratings with the within-subject factors *Target-Valence* (neutral vs. positive) and *Content* (humans vs. animals vs. food vs. sceneries) and the between-subject factor *Group* (control vs. depletion) revealed no interactions of *Content* with *Group* (*F*_3; 198_ = 0.98, *p* = .403) and no interaction of *Content* with *Target-Valence* and *Group* (*F*_3; 198_ = 2.11, *p* = .101). Again the interaction of *Target-Valence* and *Group* reported above remained stable (*F*_1; 66_ = 4.22, *p* = .044). An ANOVA of the arousal ratings with the within-subject factors *Target-Valence* (neutral vs. positive) and *Content* (humans vs. animals vs. food vs. sceneries) and the between-subject factor *Group* (control vs. depletion) revealed no interactions of *Content* with *Group* (*F*_3; 198_ = 0.85, *p* = .467) and no interaction of *Content* with *Target-Valence* and *Group* (*F*_3; 198_ = 0.29, *p* = .835). Again the interaction of *Target-Valence* and *Group* reported above remained stable (*F*_1; 66_ = 5.00, *p* = .029). In summary, there is no indication that the effect of depletion on valence and arousal was driven by specific content categories.

## Discussion

This study tested the hypothesis that ego-depletion increases approach motivation and thereby changes the emotional evaluation of appetitive cues [8]. Ego-depletion caused more positive valence ratings of neutral pictures and lower arousal ratings of positive pictures.

The manipulation was successful. The depletion variant of the text copying task was experienced as more straining but did not change mood. Neither did mood explain the change of the emotional evaluation of pictures. Furthermore, positive pictures elicited higher valence and arousal ratings as their neutral counterparts. However, we cannot rule out that floor- and ceiling effects restricted the influence of ego-depletion to the valence of neutral and the arousal of positive pictures. Finally, the control and experimental groups did not differ regarding age, gender, handedness, GPA or drug use.

In some aspects our results confirm the hypothesis by Schmeichel and colleagues [8] that the depletion of self-control increases approach motivation: In the eye of depleted people neutral pictures of humans, animals, food and sceneries became more appealing. In contrast already appealing pictures elicited less arousal, which contradicts the hypothesis of a general increase in approach motivation. Also the reduced arousal speaks against the hypothesis that depletion of self-control amplifies whatever urges or emotions the person has at the moment [21, 22].

Apart from that, our results do not contradict the study by Wagner at al. mentioned above [10] where ego-depletion did not increase emotional reactivity of the amygdala as a response to neutral or positive pictures. However, our results do not match the other study by Wagner et al. [11] that found increased neuronal responses to pictures of food but not to pictures of people or sceneries in ego-depleted chronic dieters. This suggests that a specific motivational context is necessary for ego-depletion to increase approach motivation in the respective area, e.g. the context of chronic dieting in concert with ego-depletion might increase approach motivation for food [5] or the context of habitual heavy drinking in concert with ego-depletion might increase approach motivation for alcohol [23].

Our results are limited to the conscious aspect of emotional reactions. It is possible that non-reactive measures like facial or galvanic skin responses would yield similar results as the studies by Wagner and colleagues [10, 11]. Moreover, we focused on the emotional evaluation of appetitive cues but did not assess actual approach behavior. If the participants in our study had the prospect of e.g. actual food or social reward, the effect of ego-depletion on the emotional evaluation of cues for food or affiliation might have been stronger.

In the domain of avoidance motivation, Bertrams et al. [15] documented the importance of motivational context, namely the role of anxiety. In the domain of approach motivation, future studies should manipulate the motivational context, e.g. by sexual- or food-deprivation, to investigate the effect of ego-depletion on the evaluation of appetitive cues and actual approach behavior. Nevertheless, our study provides a first step for understanding the influence of ego-depletion on the emotional evaluation of appetitive pictures.
